# CircR-loop: a novel RNA:DNA interaction on genome instability

**DOI:** 10.1186/s11658-024-00606-5

**Published:** 2024-06-14

**Authors:** Xinming Su, Yaojie Feng, Ruixiu Chen, Shiwei Duan

**Affiliations:** 1 Key Laboratory of Novel Targets and Drug Study for Neural Repair of Zhejiang Province, School of Medicine, Hangzhou City University, Hangzhou, Zhejiang China; 2 Department of Clinical Medicine, Hangzhou City University, Hangzhou, Zhejiang China; 3 Department of Nursing, Hangzhou City University, Hangzhou, Zhejiang China

**Keywords:** CircRNA, R-Loops, RNA–DNA interactions, Gene regulation, Regulatory mechanism

## Abstract

CircR-loop, a recently unearthed regulatory mechanism situated at the crossroads of circular RNA and DNA interactions, constitute a subset of R-loop. This circR-loop have emerged as a crucial player in pivotal regulatory functions within both animal and plant systems. The journey into the realm of circR-loop commenced with their discovery within the human mitochondrial genome, where they serve as critical directors of mitochondrial DNA replication. In the plant kingdom, circR-loop wield influence over processes such as alternative splicing and centromere organization, impacting the intricacies of floral development and genome stability, respectively. Their significance extends to the animal domain, where circR-loop has captured attention for their roles in cancer-related phenomena, exerting control over transcription, chromatin architecture, and orchestrating responses to DNA damage. Moreover, their involvement in nuclear export anomalies further underscores their prominence in cellular regulation. This article summarizes the important regulatory mechanisms and physiological roles of circR-loop in plants and animals, and offers a comprehensive exploration of the methodologies employed for the identification, characterization, and functional analysis of circR-loop, underscoring the pressing need for innovative approaches that can effectively distinguish them from their linear RNA counterparts while elucidating their precise functions. Lastly, the article sheds light on the challenges and opportunities that lie ahead in the field of circR-loop research, emphasizing the vital importance of continued investigations to uncover their regulatory roles and potential applications in the realm of biology. In summary, circR-loop represents a captivating and novel regulatory mechanism with broad-reaching implications spanning the realms of genetics, epigenetics, and disease biology. Their exploration opens new avenues for comprehending gene regulation and holds significant promise for future therapeutic interventions.

## Background

RNA–DNA hybrids serve as crucial intermediates in various cellular processes, including transcription, DNA replication, and DNA repair [1]. Among these, R-Loops represent a distinctive nucleic acid structure, consisting of DNA–RNA hybrids intertwined with related non-template single-stranded DNA during transcription [1]. In mammalian cells, approximately 5% of genomic regions give rise to R-Loops, with a prevalent presence in GC-rich sequences, CpG islands, and at transcription start and stop sites distinguished by GC skew [2]. R-Loops can be categorized into two types: non-pathological and pathological, contingent upon the context in which they form. Non-pathological R-Loops inherently manifest across all transcriptional processes, playing pivotal roles in gene expression regulation and chromatin structural modulation [2]. Conversely, pathological R-Loops are comparatively rare, and their excessive accumulation can profoundly impede transcription, mRNA production, induce mutagenesis, and hamper gene expression, culminating in conditions like neurodegeneration and cancer [3, 4]. Consequently, analyzing R-Loop distribution and elucidating the mechanisms regulating gene expression are of paramount importance.

Notably, RNA transcribed by RNA Polymerases I, II, and III can all engage in R-Loop formation with DNA, highlighting their versatility across diverse RNA types [1]. CircularRNA (CircRNA), a subset of non-coding RNA (ncRNA), emerges from selective splicing of one or more gene exons, facilitated by the RNA-induced silencing complex (RISC) [5]. Unlike other linear ncRNAs such as miRNA and lncRNA, circRNAs possess a distinctive structural feature wherein their 3' and 5' ends are covalently joined, creating a closed circular configuration [6]. This closed circularity enhances the stability of circRNAs, leading to their conservation across eukaryotes, specific subcellular localization, and diverse biological functions [7]. Increasingly, research indicates that circRNAs exhibit multifaceted functions, serving as transcription regulators, microRNA sponges, and even protein templates [8]. In a recent breakthrough, it has been discovered that circRNAs can also form hybrids with DNA, referred to as circR-loop, which actively participate in the regulation of biological processes in both animals and plants. This article provides an in-depth review of the mechanisms and consequences of circR-loop involvement in animals and plants, along with an overview of common experimental approaches for studying circR-loop. Furthermore, the article identifies current research limitations and proposes potential future research directions, offering a comprehensive framework and research insights for the ongoing exploration of circR-loop.

## Unveiling the regulatory significance of circR-loop: from mitochondrial mysteries to genome-wide impacts in animals and plants

### Discovery and characterization of circR-loop in mitochondria

Circular RNA–DNA hybrid, known as circR-loop, were initially identified and investigated within the human mitochondrial genome. In a pioneering study conducted in 1998 by Lee et al., it was revealed that the initiation of guide-strand mitochondrial DNA (mtDNA) synthesis in vitro necessitates the actions of RNA polymerase and RNase MRP to generate replication primers. Remarkably, these processed RNA products exhibited the ability to maintain stable base pairing with the template DNA strand, subsequently kickstarting mtDNA replication on the closed circular plasmid [9]. This unique R-Loop phenomenon was found to be situated proximal to the D-loop, a highly stable mixed g-quadruplex structure formed by the parental DNA strand and RNA transcript. This interaction ultimately governs the initial rate of DNA synthesis in human mitochondria [10, 11].

### The role of circR-loop in plant genetic regulation

For an extended period, RNA–DNA hybrids of this nature were treated as isolated instances and largely overlooked. Only in recent years have comprehensive investigations explored their regulatory roles in the realms of animals and plants. In the model plant Arabidopsis thaliana, R-Loops were discovered to be prevalent and influential in the regulation of alternative splicing (AS). Notably, circRNAs originating from SEPALLATA3 (SEP3) Exon6 exhibited a pronounced affinity for their cognate DNA sites, facilitating circR-loop formation and amplifying the accumulation of SPE3.3 variants. This, in turn, enhanced the interaction between SPE3 Exon 6 circRNA and the SPE3 gene, establishing a positive feedback loop integral to the regulation of the development of all four floral organs—calyx, petals, stamens, and pistils [12]. Additionally, R-Loop mapping in stem-differentiating xylem (SDX) hinted at the regulatory functions of various other circRNAs in AS [13]. Simultaneously, circR-loop was also identified as pivotal players in the regulation of plant centromeres. In maize, multiple circRNAs derived from centromeric retrotransposons (CRMs), also known as centromeric-derived RNAs (cenRNAs), demonstrated the ability to bind to centromeres. This activity played a pivotal role in orchestrating a conducive chromatin environment during centromere evolution, regulating the formation of maize centromeric chromatin loops [14].

### The regulatory function of circR-loop in animal cancer

In the context of animals, circR-loop functions have been intensely explored in the realm of cancer. For instance, in breast cancer (BC), the R-Loops generated by circSMARCA5 binding to its parent locus SMARCA5 were found to induce transcriptional pausing of SMARCA5 exon 15. This resulted in the downregulation of SMARCA5 gene expression and the production and degradation of truncated non-functional proteins, heightening sensitivity to cytotoxic drugs [15]. More recently, circRNA-DNA interactions were identified as pervasive throughout the genome in mixed lineage leukemia (MLL). A subset of circR-loop actively participated in promoting transcription pausing, proteasome inhibition, chromatin reorganization, and DNA fragmentation through a newly defined mechanism known as endogenous RNA-directed DNA damage (ER3D). This mechanism led to oncogenic mutations and cancer development [16]. Moreover, a study delving into the regulatory mechanism of circR-loop formation revealed that the nuclear accumulation of circRNA triggers circR-loop formation, subsequently inducing DNA damage as part of disease regulation [17]. Importantly, this nuclear export anomaly exhibited significant associations with differential Exportin4 (XPO4) expression and high circRNA binding capacity, with sensitivity influenced by tissue and circRNA species [17].

Collectively, it becomes evident that circR-loop, along with other RNA–DNA hybrids resulting from RNA–DNA interactions, share similar spatiotemporal specificity and regulatory mechanisms. Moreover, it is noteworthy that the average expression levels of circRNAs in peripheral blood surpass those of their host linear genes [15], and the hybridization efficiency of circRNAs with homologous DNA is substantially higher compared to linear RNAs [16]. This underscores the potential for a more extensive and potent regulatory role of circR-loop, thus warranting further exploration and in-depth investigation.

## Exploring the world of circR-loop: identification, characterization, and functional insights

### Identification and verification of circR-loop

As a relatively novel regulatory structure, the identification and verification system for circR-loop is still evolving. Currently, researchers often stumble upon circR-loop and their regulatory functions while delving into the mechanisms of circRNA. For instance, by investigating whether circSMARCA5 could halt SMARCA5 transcription elongation, researchers uncovered the genome-level regulatory role of circR-loop [15].

The primary prerequisite for identifying circR-loop is the ability to distinguish circRNA from linear RNA. Key enzymes in this process include RNase H and RNase R. CircRNA is typically sensitive to RNase H treatment but resistant to RNase R treatment. Consequently, employing RNase R to initiate decay on the single-stranded 3' dangling strand can effectively degrade most linear RNA, thereby purifying and enriching circRNA. Further, researchers frequently employ short-read sequencing (such as Sanger or Illumina sequencing) on RNA libraries post-rRNA removal and RNase R treatment. Alternatively, long-read sequencing methods like PacBio and Nanopore may be used. Moreover, experiments involving RNase R resistance, RT-PCR, northern blotting, and other techniques serve to validate circRNA. To gain a comprehensive understanding of circRNA, it's essential to characterize aspects such as expression levels, length distribution, GC content, and ΔG distribution. It's worth noting that nuclear abundance does not always reflect R-loop functionality [17].

In subsequent steps, researchers often combine techniques like ChIP and RIP with the sequencing methods mentioned above to capture interactions between circRNA and DNA. DNA:RNA hybrid immunoprecipitation and sequencing (DRIP-seq) represent a groundbreaking approach for R-loop identification. S9.6 is a monoclonal antibody derived from mice through immunization with phage ϕX174. It exhibits high affinity and specificity for binding to RNA:DNA hybrids [18]. DRIP-seq utilizes the S9.6 antibody, which selectively recognizes RNA:DNA hybrid strands, to enrich DNA strands containing the R-loop structure. Subsequently, R-loop analysis is conducted through sequencing [19]. Therefore, in the identification of circR-loops, DRIP-seq is typically combined with circRNA sequencing (circRNA-seq) to assess the overlap of circRNA homologous sites and to initially screen potential circR-loops [19].Typically, DRIP-seq is combined with circRNA sequencing (circRNA-seq) to assess overlap at circRNA homologous sites and preliminarily screen for potential circR-loop. By utilizing the structure-specific S9.6 monoclonal antibody, researchers can capture genome-wide R-loop structures along chromosomes via DRIP-seq and quantitatively evaluate hybridization intensity through techniques like dot-blotting and qPCR [20].

Subsequently, fluorescence in situ hybridization (FISH) enables visualization and cellular localization. However, traditional FISH faces challenges such as unstable signal quality, limiting its application [21]. Three-dimensional RNA–DNA fluorescence in situ hybridization (3D RNA/DNA-FISH), employing different probes targeting DNA and RNA, allows for optimization of hybridization conditions. It enables multi-color fluorescent labeling, offering a more intuitive and clear method to confirm the formation of circR-loops [22]. Furthermore, the integration of circular chromosome conformation capture (4C-seq) with 3D-FISH aids in elucidating the relationship between chromatin interactions and biological processes [16, 23, 24].

### Exploration of circR-loop expression and function

Finally, by integrating circRNA and R-loop characteristics, researchers employ corresponding experiments to explore differential expression and biological functions of circR-loop. R-loops have long been associated with regulating AS, promoting transcription pausing, and causing double-strand DNA breaks (DSBs) [25]. Similar functions may apply to circR-loop. To investigate their potential to regulate AS, researchers design specific primers and utilize RT-PCR. Assessment of RNAPII occupancy aids in exploring circR-loop involvement in transcriptional pausing. The ability of circR-loop to induce specific DSBs at their homologous loci is evaluated using comet assays. For downstream regulatory mechanisms, enrichment analysis following circRNA gene overexpression or knockdown serves as an initial functional assessment. To investigate their biomarker potential, researchers often employ serum samples in conjunction with Real-time PCR and Northern blotting for validation. To understand their in vivo and in vitro effects, Loss of Function and Gain of Function experiments are conducted using appropriate models to verify the functions of candidate circR-loop.

In conclusion, current research methods for circR-loop closely resemble those for traditional R-loops, emphasizing the interconnectedness of structure and function. The unique RNA structure of circR-loop may confer distinct regulatory functions and strengths compared to other types of R-loops. Future research should focus on elucidating circR-loops' functions and developing specific characterization methods, particularly to differentiate their action times and intensities from traditional linear RNA. Simultaneously, advancing more efficient and accurate technical approaches, such as utilizing enhanced probes for improved spatial resolution, holds significant importance in driving research advancements in the circRNA field. Additionally, investigating how circRNA functions through circR-loop requires innovative conceptual approaches and experimental methods.

## Unveiling the enigma of circR-loop: challenges and opportunities in the study of a novel regulatory mechanism

### Identification and characterization of circR-loop

As a universal and extensively studied regulatory mechanism, the structure and function of R-loops have been thoroughly explored. However, the discovery of circR-loop, formed through interactions between circRNA and DNA, is a relatively recent development, with only a limited number of dedicated studies to date [13, 16]. This scarcity of research may be attributed to the challenges in identifying and distinguishing circR-loop. While circRNA was observed as early as 1979, its unique structure made it challenging to identify, often leading to confusion with linear RNA or outright dismissal as an aberrant sequence. It wasn't until 2013, with advancements in sequencing technology, that circRNA gained recognition and widespread study [26, 27]. Currently, research on circR-loop faces similar obstacles, as distinguishing circR-loop from other R-loops primarily relies on RNase H and RNase R processing or the speculative overlap of genomic sites. Novel methods for isolating, purifying, amplifying, and stabilizing circR-loop have yet to emerge, and standardized identification and classification criteria remain underdeveloped.

It is heartening to witness the development of breakthrough characterization technologies, which often offer superior genome coverage and enhanced detection accuracy compared to traditional methods. A notable example is the recent development of Cross-linked Poly(A) Pulldown RNase R sequencing (CLiPPR-seq) technology by Amaresh C. Panda and colleagues. This innovative approach employs 4′-aminomethyltrioxsalen hydrochloride (AMT)-mediated RNA-RNA double-stranded cross-linking treatment for enriching circular RNA and subsequent deep sequencing. By applying CLiPPR-seq, hundreds of circRNAs interacting with mRNA were identified across three different intact cell types, including βTC6, C2C12, and HeLa [28]. The continued refinement of this pioneering technique holds promise for uncovering unexplored circR-loops that potentially regulate gene expression.

The progression of sequencing technology is pivotal for high-throughput screening and identification of circR-loop, and future research must establish classification standards and innovative detection techniques to facilitate comprehensive mapping, laying the groundwork for subsequent investigations. Additionally, a more practical avenue for improvement involves integrating existing technologies and designing approaches based on specific sites or structures involved in the biological processes of R-loop formation and resolution. This approach would target genomic single-stranded DNA (ssDNA) exposure using techniques such as KAS-seq, CUT&TAG, and others, which would be combined and enhanced with circRNA characterization technology. This especially includes long-read sequencing detection based on third-generation sequencing technology, allowing for precise localization of circR-loop structures.

### Establishing a standardized nomenclature for circR-loop

Standardization of the naming of newly discovered structures is crucial for promoting academic exchange and cooperation. However, due to limited research, there is currently a lack of unified nomenclature and classification standards for the R-loops formed by this type of circRNA, further complicating its definition and classification. Therefore, we propose the use of "circR-loop" as a unified name for this special structure. The prefix "circ" signifies that the RNA molecule involved is circRNA with a covalently closed circular structure, imparting unique stability and functional properties compared to linear RNA. The "R-loop" section emphasizes the distinctive hybrid structure formed between the RNA part and DNA, a phenomenon well-studied and confirmed previously [29, 30].

### The origin, production and degradation mechanism of circR-loop

Meanwhile, critical biological properties such as the origins and mechanisms of action of circR-loop remain largely uncharted territory. While the formation mechanism and influencing factors of circR-loop are assumed to parallel those of traditional R-loops, specific mechanisms have yet to be uncovered and elucidated. Notably, while circR-loop structure has been explored in the mitochondrial genome, its structure within the nucleus remains unexplored. Given its status as an RNA–DNA interaction, the nuclear structure of circR-loop may possess broader relevance. Understanding the origin and structure of circR-loop is crucial, as it can shed light on the specificity of their regulatory functions both within and between species, warranting in-depth exploration as a priority.

As a type of ncRNA, circR-loops are theoretically formed by the hybridization between circRNA and distant DNA strands through trans interaction [1]. However, the specific formation process and mechanism of circR-loops are still not fully characterized. Future research can focus on the unique spatial structure of circRNA and R-loops, exploring the characteristic local chromatin 3D conformation and sequences (such as Alu and its derivatives) before circR-loop formation. This investigation will shed light on the novel mechanism underlying the formation of this specialized R-loop structure [31–33].

Moreover, the stabilization and degradation processes following circR-loop formation are also of great interest. Due to its covalently closed-loop structure, circRNA is typically more stable than linear RNA, resisting degradation by RNases and clearance by the body's natural immunity [34]. Various factors, including endonuclease activity, structural changes, and chemical substances, participate in circRNA degradation [35]. Conversely, the stability of R-loops is known to be influenced by factors such as transcription, GC content, and the presence of G-quadruplex structures [36–38]. Additionally, RNA binding and chromatin modification factors, along with helicases/translocases, topoisomerases, and replication/repair factors, collaborate to either form or eliminate R-loops [39, 40]. Given that nuclear accumulation of circRNAs can promote the formation of deleterious R-loops [17], further exploration of the degradation mechanism of circR-loops and associated components using advanced characterization technologies, such as microfluidics and sequencing, is essential. Understanding these mechanisms is crucial for developing related technologies to maintain cellular homeostasis and facilitate the biological functions of circR-loops [35].

### CircR-loop’s unique and complex regulatory functions

Currently, the regulatory mechanism of circR-loop remains inadequately studied. Previous research has shown that regulatory R-loops play a crucial role in maintaining genome stability by modulating transcription activity, replication, recombination, centromere function, and DNA editing [29]. Additionally, circRNAs have been identified as regulators of gene expression through mechanisms such as miRNA or protein sponge adsorption and interaction with synthetic peptides [34]. Importantly, circRNAs can independently regulate genome stability or collaborate with other proteins in this process [34]. Consequently, it is anticipated that future research will focus on elucidating the role of circR-loop in regulating genome stability. Furthermore, given the widespread distribution of circRNAs and R-loops, it is likely that circR-loops are involved in regulating downstream gene expression through diverse mechanisms. Additionally, owing to the exceptional stability of circRNA, its regulatory mechanisms and capabilities likely diverge from those of other R-loops, necessitating further profound inquiry. While the intrinsic regulatory function of R-loops operates at the epigenetic level, the regulatory mechanisms of circR-loop, although conceptually akin, may entail unique attributes. Future research should initially develop methods tailored to the structure and characteristics of circR-loop based on existing experiments, thereby distinguishing the specific regulatory functions of circR-loop. Furthermore, when investigating circRNA function, circR-loop should be considered as a potential regulatory mechanism, extending beyond the study of ceRNA mechanisms.

### The potential role of circR-loop in human disease

The potential impact of R-loops and circR-loops in human diseases cannot be underestimated. Dysregulated dynamics of R-loops formed by multiple linear RNAs have been implicated in DNA damage and genome instability, closely associated with diseases like cancer and autoimmune disorders [37]. In cancer, R-loop accumulation, influenced by various factors, drives DNA damage and replication stress [41, 42]. In autoimmune conditions such as Aicardi-Goutières syndrome (AGS), R-loops may contribute to disease pathogenesis by altering gene expression or reactivating retromers [43]. circRNAs have been implicated in the regulation of diverse diseases, including cancer, cardiovascular, neurological, and infectious diseases [8]. Although the regulatory role of circR-loops has been predominantly studied in cancer, with less exploration in other diseases, a recent study demonstrated that circLrch3 forms a circR-loop by binding to specific DNA sequences within the promoter of its host gene Lrch3. This promotes chromatin activation and DNA demethylation, enhancing Lrch3 transcriptional activity and leading to apoptosis of pulmonary artery smooth muscle cells [44]. Given the broad regulatory potential of circR-loop components in human diseases, further exploration across various disease types and sample cohorts is warranted to expand our understanding of the functional landscape of circRNA.

### The potential of circR-loop in clinical translational research

The distinctive expression profile of circRNA presents significant potential as biomarkers, therapeutic agents, and drug targets. As a subtype of circRNA, circR-loop possesses a distinct spatial and sequence structure, offering broad clinical applications. However, clinical translation research on circR-loop remains nascent. Future exploration should focus on elucidating circR-loop subtypes, verifying structural conservation, species specificity, and half-life. Moreover, developing risk assessment models based on circR-loop for various diseases and validating its efficacy and stability as an emerging biomarker through large-scale clinical trials is imperative [5].

In terms of therapeutics, circRNA exhibits high stability, unique folding, and low immunogenicity, sparking interest in circRNA-based treatments. Novel RNA aptamers and protein translation vectors have shown promise in both in vivo and in vitro settings. These therapeutic modalities may artificially modulate circR-loop to regulate gene expression, thereby influencing cellular behavior [8, 45]. By targeting specific binding sites presented by circR-loop, these therapies may regulate the structural stability of circR-loop, akin to competitive endogenous RNA (ceRNA) mechanisms. This potential underscores the transformation of circRNAs into effective tumor treatments. However, circRNA-based aptamer therapy remains conceptual, facing challenges in molecular optimization design and mechanistic elucidation (46).

With ongoing advancements in basic circR-loop research, the scope of research methods continues to expand and optimize. Simultaneously, platforms for in vitro transcription, cyclization, and delivery systems are maturing, auguring clearer prospects for the clinical medical transformation of circR-loop.

## Conclusions

**Fig. 1:**
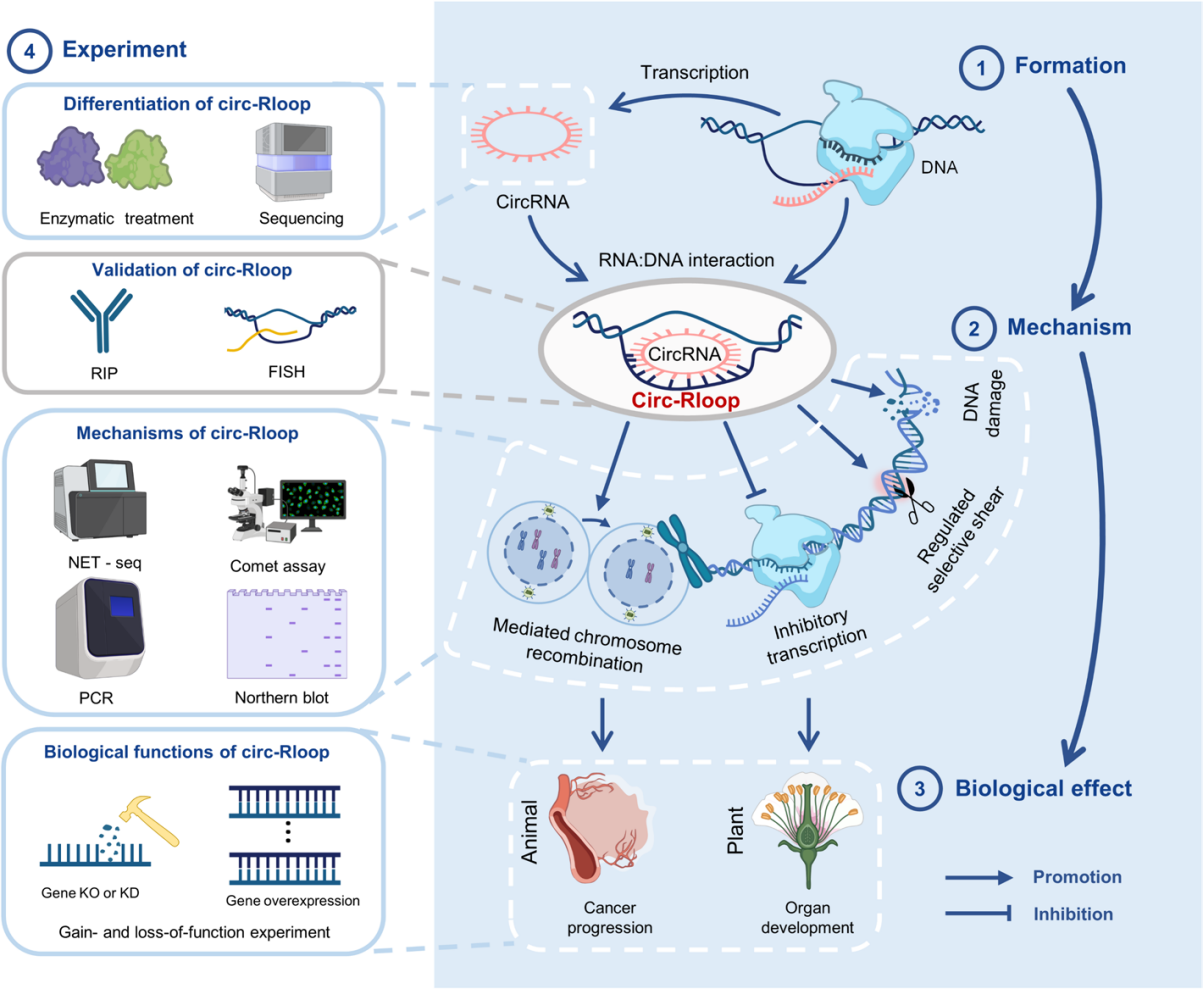
Decoding circR-loop: from transcription regulation to organ development and cancer progression. CircRNAs, transcribed from DNA, can engage with the sequence of their parent genes during RNA:DNA interactions, forming circR-loop when the transcription bubble of the parent gene is formed. Distinguishing circRNAs from linear genes typically involves a combination of enzymatic treatment and sequencing methods. Meanwhile, the characterization of circR-loop is predominantly conducted through DRIP technology and FISH coupled with RIP sequencing. circR-loop plays pivotal roles in inhibiting transcription, regulating alternative splicing, mediating chromatin reorganization, and DNA damage, as verified through corresponding phenotypic experiments. Presently, circR-loop has been predominantly identified in plants, where they mediate organ development. In animals, they are primarily implicated in the regulation of cancer progression. These effects are often validated through gain- and loss-of-function experiments, providing valuable insights into their functional significance. CircRNAs, circularRNAs; DRIP, DNA:RNA hybrid immunoprecipitation; FISH, fluorescence in situ hybridization; RIP, RNA binding protein immunoprecipitation

CircR-loop, as a novel regulatory mechanism stemming from the interplay between circRNA and DNA, may wield far-reaching regulatory roles in animals and plants (Fig. 1). In-depth exploration of their origin, structure, and mechanism is imperative, potentially offering insights into unresolved biological phenomena and establishing a foundation for targeted intervention methods in the future.

## Data Availability

Not applicable.

## Abbreviations

4C-seq: Circular chromosome conformation capture sequencing
AMT: 4′-Aminomethyltrioxsalen hydrochloride
AS: Alternative splicing
BC: Breast cancer
cenRNAs: Centromeric-derived RNAs
circRNA: CircularRNA
circRNA-seq: CircRNA sequencing
CLiPPR-seq: Cross-linked Poly(A) Pulldown RNase R sequencing
CRMs: Centromeric retrotransposons
DRIP-seq: DNA:RNA hybrid immunoprecipitation and sequencing
DSBs: Double-strand DNA breaks
ER3D: Endogenous RNA-directed DNA damage
FISH: Fluorescence in situ hybridization
MLL: Mixed lineage leukemia
mtDNA: Mitochondrial DNA
ncRNA: Non-coding RNA
RISC: RNA-induced silencing complex
SDX: Stem-differentiating xylem
SEP3: SEPALLATA3
ssDNA: Single-stranded DNA
XPO4: Exportin4

## References

[1] Petermann E, Lan L, Zou L (2022). Sources, resolution and physiological relevance of R-loops and RNA-DNA hybrids. Nat Rev Mol Cell Biol.

[2] Li X, Zhang JL, Lei YN, Liu XQ, Xue W, Zhang Y (2021). Linking circular intronic RNA degradation and function in transcription by RNase H1. Sci China Life Sci.

[3] Chakraborty P (2020). New insight into the biology of R-loops. Mutat Res.

[4] Petermann E, Lan L, Zou L (2022). Author correction: sources, resolution and physiological relevance of R-loops and RNA-DNA hybrids. Nat Rev Mol Cell Biol.

[5] Kristensen LS, Jakobsen T, Hager H, Kjems J (2022). The emerging roles of circRNAs in cancer and oncology. Nat Rev Clin Oncol.

[6] Liang Y, Liu N, Yang L, Tang J, Wang Y, Mei M (2021). A brief review of circRNA biogenesis, detection, and function. Curr Genomics.

[7] Misir S, Wu N, Yang BB (2022). Specific expression and functions of circular RNAs. Cell Death Differ.

[8] Zhou WY, Cai ZR, Liu J, Wang DS, Ju HQ, Xu RH (2020). Circular RNA: metabolism, functions and interactions with proteins. Mol Cancer.

[9] Lee DY, Clayton DA (1998). Initiation of mitochondrial DNA replication by transcription and R-loop processing. J Biol Chem.

[10] Clayton DA (2000). Transcription and replication of mitochondrial DNA. Hum Reprod.

[11] Wanrooij PH, Uhler JP, Shi Y, Westerlund F, Falkenberg M, Gustafsson CM (2012). A hybrid G-quadruplex structure formed between RNA and DNA explains the extraordinary stability of the mitochondrial R-loop. Nucleic Acids Res.

[12] Conn VM, Hugouvieux V, Nayak A, Conos SA, Capovilla G, Cildir G (2017). A circRNA from SEPALLATA3 regulates splicing of its cognate mRNA through R-loop formation. Nat Plants.

[13] Liu X, Gao Y, Liao J, Miao M, Chen K, Xi F (2021). Genome-wide profiling of circular RNAs, alternative splicing, and R-loops in stem-differentiating xylem of Populus trichocarpa. J Integr Plant Biol.

[14] Liu Y, Su H, Zhang J, Liu Y, Feng C, Han F (2020). Back-spliced RNA from retrotransposon binds to centromere and regulates centromeric chromatin loops in maize. PLoS Biol.

[15] Xu X, Zhang J, Tian Y, Gao Y, Dong X, Chen W (2020). CircRNA inhibits DNA damage repair by interacting with host gene. Mol Cancer.

[16] Conn VM, Gabryelska M, Toubia J, Kirk K, Gantley L, Powell JA (2023). Circular RNAs drive oncogenic chromosomal translocations within the MLL recombinome in leukemia. Cancer Cell.

[17] Chen L, Wang Y, Lin J, Song Z, Wang Q, Zhao W (2022). Exportin 4 depletion leads to nuclear accumulation of a subset of circular RNAs. Nat Commun.

[18] Wei E, Bou-Nader C, Perry ML, Fattah R, Zhang J, Leppla SH (2023). S9.6 antibody-enzyme conjugates for the detection of DNA-RNA hybrids. Bioconjug Chem.

[19] Chédin F, Hartono SR, Sanz LA, Vanoosthuyse V (2021). Best practices for the visualization, mapping, and manipulation of R-loops. EMBO J.

[20] Ramirez P, Crouch RJ, Cheung VG, Grunseich C (2021). R-loop analysis by dot-blot. J Vis Exp.

[21] Bejugam PR, Das A, Panda AC (2020). Seeing is believing: visualizing circular RNAs. Noncoding RNA..

[22] Marasca F, Cortesi A, Manganaro L, Bodega B (2020). 3D multicolor DNA FISH tool to study nuclear architecture in human primary cells. J Vis Exp.

[23] Matelot M, Noordermeer D (2016). Determination of high-resolution 3D chromatin organization using circular chromosome conformation capture (4C-seq). Methods Mol Biol.

[24] Zhao Z, Tavoosidana G, Sjölinder M, Göndör A, Mariano P, Wang S (2006). Circular chromosome conformation capture (4C) uncovers extensive networks of epigenetically regulated intra- and interchromosomal interactions. Nat Genet.

[25] Gambelli A, Ferrando A, Boncristiani C, Schoeftner S (2023). Regulation and function of R-loops at repetitive elements. Biochimie.

[26] Memczak S, Jens M, Elefsinioti A, Torti F, Krueger J, Rybak A (2013). Circular RNAs are a large class of animal RNAs with regulatory potency. Nature.

[27] Hansen TB, Jensen TI, Clausen BH, Bramsen JB, Finsen B, Damgaard CK (2013). Natural RNA circles function as efficient microRNA sponges. Nature.

[28] Singh S, Shyamal S, Das A, Panda AC (2024). Global identification of mRNA-interacting circular RNAs by CLiPPR-Seq. Nucleic Acids Res.

[29] Niehrs C, Luke B (2020). Regulatory R-loops as facilitators of gene expression and genome stability. Nat Rev Mol Cell Biol.

[30] Hegazy YA, Fernando CM, Tran EJ (2020). The balancing act of R-loop biology: the good, the bad, and the ugly. J Biol Chem.

[31] Ariel F, Lucero L, Christ A, Mammarella MF, Jegu T, Veluchamy A (2020). R-loop mediated trans action of the APOLO long noncoding RNA. Mol Cell.

[32] Bai X, Li F, Zhang Z (2021). A hypothetical model of trans-acting R-loops-mediated promoter-enhancer interactions by Alu elements. J Genet Genomics.

[33] Toriumi K, Tsukahara T, Hanai R (2013). R-loop formation in trans at an AGGAG repeat. J Nucleic Acids.

[34] Liu CX, Chen LL (2022). Circular RNAs: characterization, cellular roles, and applications. Cell.

[35] Ren L, Jiang Q, Mo L, Tan L, Dong Q, Meng L (2022). Mechanisms of circular RNA degradation. Commun Biol.

[36] Aguilera A, García-Muse T (2012). R loops: from transcription byproducts to threats to genome stability. Mol Cell.

[37] Crossley MP, Bocek M, Cimprich KA (2019). R-loops as cellular regulators and genomic threats. Mol Cell.

[38] García-Muse T, Aguilera A (2019). R loops: from physiological to pathological roles. Cell.

[39] Bonnet A, Grosso AR, Elkaoutari A, Coleno E, Presle A, Sridhara SC (2017). Introns protect eukaryotic genomes from transcription-associated genetic instability. Mol Cell.

[40] Santos-Pereira JM, Aguilera A (2015). R loops: new modulators of genome dynamics and function. Nat Rev Genet.

[41] Stork CT, Bocek M, Crossley MP, Sollier J, Sanz LA, Chédin F (2016). Co-transcriptional R-loops are the main cause of estrogen-induced DNA damage. Elife.

[42] Kotsantis P, Silva LM, Irmscher S, Jones RM, Folkes L, Gromak N (2016). Increased global transcription activity as a mechanism of replication stress in cancer. Nat Commun.

[43] Park K, Ryoo J, Jeong H, Kim M, Lee S, Hwang SY (2021). Aicardi-Goutières syndrome-associated gene SAMHD1 preserves genome integrity by preventing R-loop formation at transcription-replication conflict regions. PLoS Genet.

[44] Liu H, Jiang Y, Shi R, Hao Y, Li M, Bai J (2024). Super enhancer-associated circRNA-circLrch3 regulates hypoxia-induced pulmonary arterial smooth muscle cells pyroptosis by formation of R-loop with host gene. Int J Biol Macromol.

[45] Chen LL (2020). The expanding regulatory mechanisms and cellular functions of circular RNAs. Nat Rev Mol Cell Biol.

[46] He AT, Liu J, Li F, Yang BB (2021). Targeting circular RNAs as a therapeutic approach: current strategies and challenges. Signal Transduct Target Ther.

